# A gender-integrated biopsychosocial model to understand cardiovascular risk in women working under environmental hazards: the case of chronic intermittent hypoxia

**DOI:** 10.3389/fpubh.2025.1672503

**Published:** 2025-09-22

**Authors:** Morin Lang, Chiara Cazzuffi, Ginés Viscor, Johana Soto-Sánchez

**Affiliations:** ^1^Department of Physical Therapy, Faculty of Medicine, University of Chile, Santiago, Chile; ^2^Faculty of Medicine, School of Kinesiology, Universidad Finis Terrae, Santiago, Chile; ^3^Centro de Economía y Políticas Sociales (CEAS), Facultad de Ciencias Sociales y Artes, Universidad Mayor, Santiago, Chile; ^4^Centro de Biomedicina, Laboratorio de Actividad Física, Ejercicio y Salud, Santiago, Chile; ^5^Department of Cell Biology, Physiology and Immunology, Faculty of Biology, Universitat de Barcelona, Barcelona, Spain; ^6^Centro de Biomedicina, Laboratorio de Actividad Física, Ejercicio y Salud, Universidad Mayor, Huechuraba, Chile

**Keywords:** high-altitude physiology, risk factors, chronic intermittent hypoxia, women's health, occupational health

## Abstract

Women working in high-altitude (HA) mining environments are exposed to chronic intermittent hypoxia (CIH), a physiological stressor resulting from rotating work shifts between sea level and elevations typically above 3,000 meters above sea level (m.a.s.l). CIH involves repeated exposure to hypobaric hypoxia, imposing significant biological, psychological, and social demands. Despite increasing female participation in the mining sector, the long-term cardiovascular risks specific to women in these conditions remain poorly characterized. This mini-review introduces the Gender-Integrated Biopsychosocial Model (GBM). This conceptual framework integrates biological, psychological, and social dimensions to examine how sex hormones, emotional burden, and gendered occupational exposures shape cardiovascular and autonomic responses to CIH. Unlike existing models that primarily reflect male physiology, the GBM emphasizes the role of natural cycling hormonal fluctuations, contraceptive use, menopause, and structural inequities in modulating cardiovascular adaptation. By advancing a multidimensional, sex and gender informed perspective, the GBM offers a novel approach to understanding women's health in extreme environments and highlights the need for occupational and environmental physiology research to recognize gender not merely as a biological variable, but as a determinant of cardiovascular risk. This article contributes to the understanding of environmental and occupational hazards in extreme workplaces by introducing an integrative model that addresses gendered exposures and physiological responses under chronic intermittent hypoxia.

## Introduction

Women working in high-altitude (HA) mining operations in Chile are exposed to intricate physiological, psychological, and social stressors, attributable to the 7 × 7-day rotating shift schedule. This schedule alternates between work at elevations exceeding 3,000 m.a.s.l. and rest at sea level. Such a pattern serves as an empirical model of chronic intermittent hypobaric hypoxia (CIH), a condition acknowledged in Decree No. 28 (1). Regarding its hazards to the cardiopulmonary and neurological systems, research involving male miners has demonstrated immediate elevations in blood pressure (BP) and heart rate (HR), along with a reduction in oxygen saturation during initial exposure at high altitude (HA) (2), with numerous cases surpassing diagnostic hypertension thresholds under 24-h ambulatory monitoring (3). However, these findings have seldom been generalized to women, as they may exhibit different cardiovascular responses owing to hormonal, emotional, and social factors.

Although traditional cardiovascular risk factors (e.g., hypertension, obesity, smoking, physical inactivity) are prevalent among miners (4). Research has not sufficiently addressed how these risks manifest in women under sex-specific exposures. Factors such as menstrual cycle, contraceptive use, work-family conflict, perceived discrimination, and symbolic violence in male-dominated settings may exacerbate cardiovascular strain. Despite increased female participation in mining (15% as of 2022), equity gaps persist, especially in access to data on workload, work shift patterns, and job roles (5, 6).

To address these intersecting challenges, we introduce a Gender-Integrated Biopsychosocial Model (GBM), grounded in George L. Engel's original framework (7), and adapted to the environmental and gendered context of high-altitude mining. This model conceptualizes cardiovascular risk as a dynamic product of: (a) biological factors: hormonal regulation and reproductive history, cardiovascular and cardiac autonomic responses, and physical workload and nutritional status under hypoxia; (b) psychological factors: mental workload, emotional strain and coping perception, and circadian and neuroendocrine stress; and (c) social factors: work-family balance, workplace dynamics, and structural barriers to women's health.

**Figure 1:**
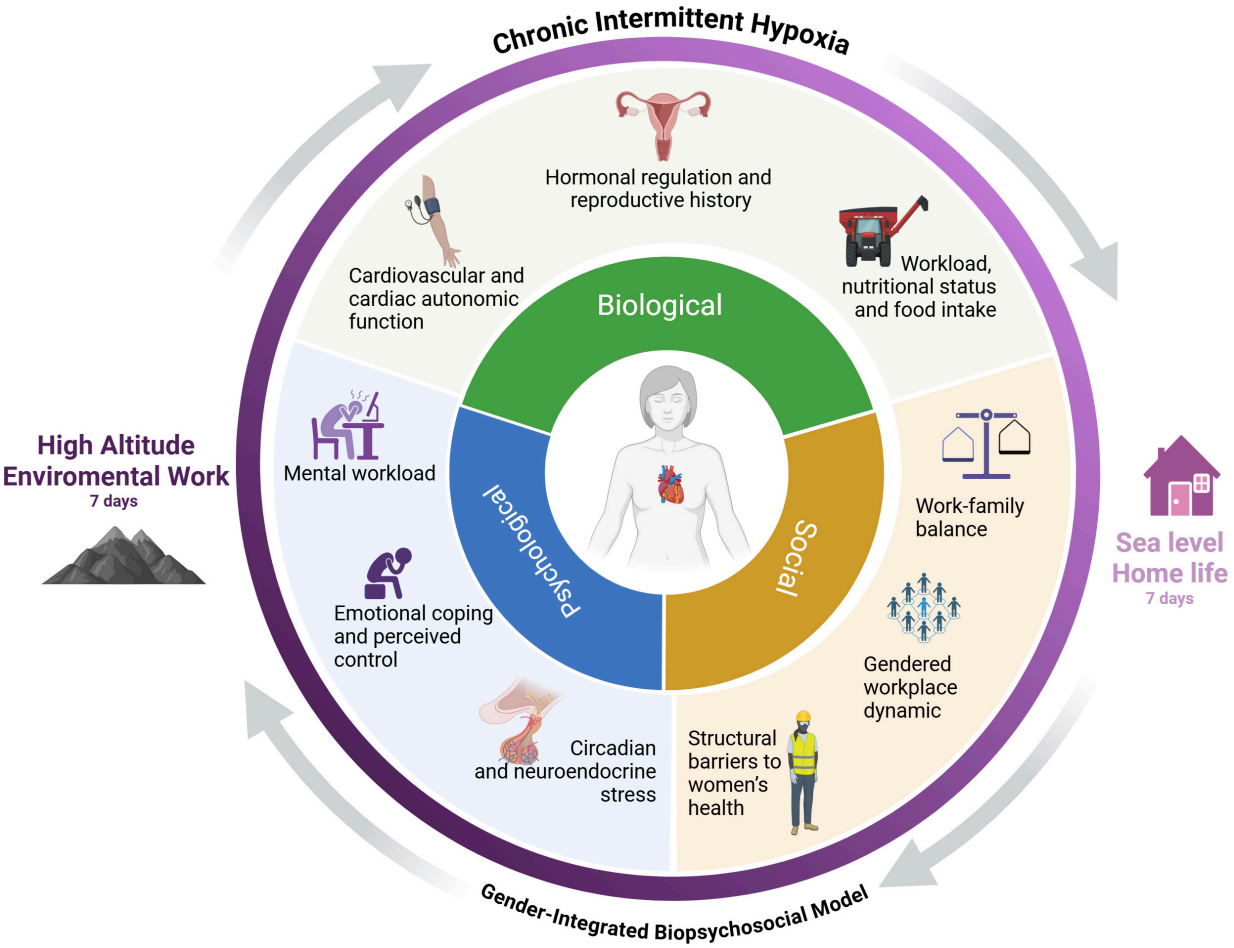
Gender-Integrated Biopsychosocial Model (GBM) of cardiovascular risk in women exposed to chronic intermittent hypoxia. This conceptual model illustrates three interrelated dimensions: Biological, Psychological, and Social, each comprising key subcomponents that contribute to cardiovascular risk in women working under conditions of chronic intermittent hypoxia (CIH), such as high-altitude mining. Biological factors include hormonal regulation and reproductive history, cardiovascular and autonomic responses, workload, nutritional status, and food intake. Psychological factors involve mental workload, emotional strain, and circadian or neuroendocrine stress. Social factors encompass work–family balance, gendered workplace dynamics, and structural barriers to women's health. Created in https://BioRender.com.

The GBM provides a novel interdisciplinary framework for evaluating how these dimensions interact under conditions of CIH. It also establishes a foundation for developing interventions aimed at improving cardiovascular health from a gender-equitable and context-sensitive perspective. The GBM synthesizes biological, psychological, and social determinants to explain cardiovascular risk in women exposed to CIH, providing a structured framework for the review, as illustrated in Figure 1.

The aim of this mini-review is to introduce and contextualize the GBM as a guiding framework for future research design, facilitating a deeper understanding of cardiovascular risk in women exposed to CIH, with a particular focus on sex- and gender-specific mechanisms that are still critically understudied within environmental and occupational physiology. Table 1 consolidates the main sex-based (biological) and gender-based (sociocultural) determinants of cardiovascular risk encompassed within the GBM framework.

**Table 1:** Sex-based (biological) and gender-based (sociocultural) determinants of cardiovascular risk in women exposed to chronic intermittent hypoxia according to the Gender-Integrated Biopsychosocial Model (GBM).

**GBM dimension**	**Sex-based determinants (Biological)**	**Gender-based determinants (sociocultural)**
Biological	• Hormonal regulation and reproductive history (menstrual cycle phase, pregnancy, menopause, contraceptive use) (12, 27, 29). • Cardiovascular and autonomic responses to hypoxia (blood pressure regulation, heart rate variability) (3, 30, 33). • Body composition and fat distribution (visceral adipose tissue, waist circumference) (33, 34). • Nutritional status and micronutrient balance (iron homeostasis) (38, 40).	–
Psychological	• Neuroendocrine stress reactivity (HPA axis, cortisol response) (53). • Sleep and circadian rhythm sensitivity to hypoxia (51).	• Mental workload related to job demands and decision-making latitude (46, 50). • Emotional coping and perceived control (influenced by caregiving responsibilities, social support) (47, 49). • Sleep disruption linked to work–family conflict (59, 61).
Social	–	• Work–family balance and caregiving roles (48, 62). • Gendered workplace dynamics in male-dominated environments (e.g., discrimination, overperformance pressure) (63, 67). • Structural barriers to women's health (lack of gender-specific PPE, menstrual hygiene facilities, pregnancy/menopause support) (54, 67).

## Biological dimension

Working at high altitudes encompasses a broad spectrum of risks, both climatic, ranging from frostbite to heat stroke, and occupational, including fatigue and circadian rhythm disturbances, which can affect individuals irrespective of gender or age. The focus will be on how sex and gender differences markedly influence cardiovascular biology and disease progression. In specific underlying conditions such as hypertension, obesity, and diabetes, women frequently display unique epidemiological profiles; however, clinical guidelines continue to underrepresent sex-specific considerations in diagnosis and treatment (8–10). In HA mining environments, women continue to be underrepresented in physiological research, thereby constraining our comprehension of their adaptations to CIH (11). In the GBM model (Figure 1), the biological dimension is organized into three primary subcomponents: hormonal status and reproductive history, cardiovascular and autonomic function, as well as physical workload, nutritional status, and food intake. These elements are further elaborated upon in this section under more specific thematic subtitles.

## Women's cardiovascular risk profile

Cardiovascular (CV) risk profiles differ between women and men due to sex-specific factors. Women face additional risks related to their reproductive history, such as adverse pregnancy outcomes, including preeclampsia and gestational diabetes (12, 13). Conditions such as polycystic ovary syndrome, early menopause, and autoimmune diseases further increase the risk (14, 15). Traditional risk factors such as diabetes and smoking confer greater relative risks in women (16, 17), and the menopause transition accelerates vascular and metabolic changes (18). Recognizing these factors facilitates the early identification of women at high risk and enables the implementation of targeted prevention strategies (19). Their presence may exacerbate cardiovascular strain in combination with environmental stressors such as CIH.

## Hormonal regulation and cardiovascular response

Hormonal regulation plays a crucial role in cardiovascular physiology, autonomic function, and body composition, directly impacting cardiovascular risk. Estradiol (E2) facilitates vasodilation via nitric oxide (NO) synthesis, thereby enhancing endothelial function and reducing blood pressure (20–22). Conversely, progesterone (P4) and androgens promote sodium retention, sympathetic nervous system activity, autonomic imbalance, and an increase in visceral adiposity and waist circumference, thereby collectively elevating cardiovascular risk (23–26). E2 has demonstrated vasoprotective and hypotensive effects across multiple vascular beds (22, 27, 28). In eumenorrheic women, hormonal fluctuations throughout the menstrual cycle substantially influence cardiovascular autonomic regulation. The follicular phase, characterized by elevated (E2), favors parasympathetic dominance, resulting in relative bradycardia and hypotension. Conversely, the luteal phase, marked by increased (P4), encourages sympathetic activation, leading to higher blood pressure (BP), increased HR, reduced heart rate variability (HRV), and heightened cardiovascular strain (27, 29–31). Furthermore, androgen-dominant conditions, such as the use of oral contraceptives (OCs) exhibiting high androgenic activity or menopause without the administration of hormone replacement therapy (HRT), are correlated with estrogen deprivation, persistent sympathetic activation, reduced vasodilation, and increased autonomic imbalance (32).

Conversely, women generally demonstrate a higher total fat mass at a specific body mass index (BMI), primarily characterized by gluteofemoral adipose tissue accumulation. In comparison, men exhibit greater fat-free mass (FFM) but possess a higher amount of visceral adipose tissue (VAT), which is a significant risk factor for CVD (33). Nonetheless, increased androgen levels in females (such as obesity, PCOS, and post-menopause) may modify their typical fat deposition patterns, increasing VAT and waist circumference (WC), thereby increasing cardiovascular risk (26, 34–36).

**Figure 2:**
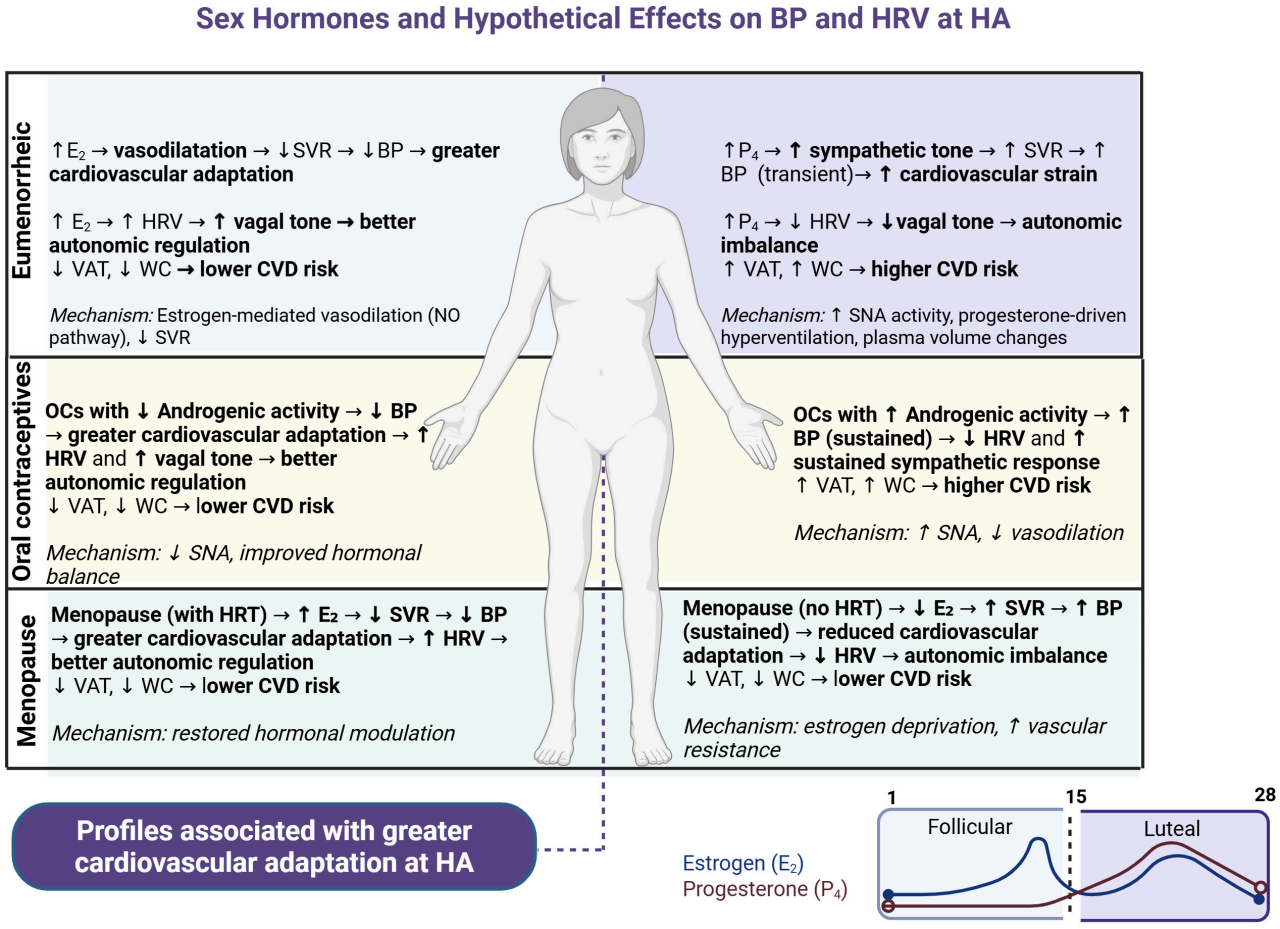
Hypothetical effects of sex hormones on Blood Pressure (BP), Heart Rate Variability (HRV), and body composition in women exposed to Chronic Intermittent Hypoxia (CIH). This diagram illustrates proposed cardiovascular, autonomic, and body composition responses across different hormonal profiles in women. Estrogen-dominant states [e.g., follicular phase, low-androgenic oral contraceptives (OCs), menopause with hormone replacement therapy (HRT)] promote vasodilation, enhanced HRV, decreased visceral adipose tissue (VAT), and waist circumference (WC), collectively contributing to improved cardiovascular adaptation. Conversely, progesterone-dominant or androgenic conditions (e.g., luteal phase, androgenic OCs, menopause without HRT) increase sympathetic nervous activity, reduce HRV, and elevate VAT and WC, thereby increasing cardiovascular strain. “Transient” BP changes refer to short-term elevations typically observed during luteal-phase hormonal shifts. “Sustained” BP indicates chronic or long-lasting elevations associated with persistent hormonal environments in women residing at high altitude.

Therefore, it is essential to comprehensively consider hormonal profiles, contraceptive use, and menopausal status when evaluating cardiovascular risk, particularly in women exposed to CIH at HA. We hypothesize that variations in hormonal status substantially influence cardiovascular and autonomic responses in women subjected to CIH. States characterized by estrogen dominance are presumed to promote cardiovascular adaptation, whereas states dominated by progesterone and androgens may elevate cardiovascular strain during exposure to hypobaric hypoxia. Figure 2 summarizes the hypothetical responses corresponding to hormonal status relevant to high-altitude occupational exposure in women.

## Workload, nutrition, and food intake sex-based responses to hypoxia

Workload and nutritional status critically interact with hormonal and reproductive factors to modulate cardiovascular and reproductive strain in women working at HA. Mining activities generally entail extended periods of standing, manual exertion, and continuous physical effort in cold, dry, and hypoxic environments. These operations impose considerable physiological stress owing to decreased oxygen availability resulting from a hypobaric setting. In comparison to men, women consistently demonstrate higher HR, increased ventilatory responses, and greater perceived exertion at comparable levels of workload (30, 37). These characteristics are further influenced by the menstrual cycle, wherein increased P4 levels during the luteal phase enhance sympathetic activity and ventilatory drive, thereby intensifying cardiovascular burden (27).

Nutritional status considerably impacts physiological adaptation to HA. Inadequate energy intake and deficiencies in micronutrients, especially iron, can compromise vascular function, intensify oxidative stress, and disturb hormonal equilibrium. Women residing at altitude are susceptible to iron depletion, which adversely affects both cardiovascular performance and reproductive health (38, 39). Furthermore, the menstrual cycle influences iron homeostasis by connecting hormonal fluctuations to nutrient metabolism (38, 40). Both iron deficiency and overload are associated with adverse reproductive outcomes, underscoring the sensitivity of female fertility to nutritional balance (41, 42). Importantly, these nutritional factors align with hormonal influences on body composition, particularly leading to increased VAT and WC in androgenic profiles (obesity, PCOS, post-menopause). This central fat redistribution directly contributes to heightened cardiovascular risk, as previously described (26, 34).

Despite these well-established links, current occupational health frameworks seldom account for sex-specific nutritional, cardiovascular, and hormonal interactions. An integrated approach is necessary to safeguard the wellbeing and optimize the performance of women operating in extreme environments, such as high-altitude mining.

Hypertension, a prominent cardiovascular risk factor among the Chilean population, has been extensively documented in male miners exposed to CIH (3, 43). Nevertheless, limited research has assessed the interaction between workload intensity and nutritional status with sex-specific mechanisms in influencing blood pressure regulation among women engaged in employment HA.

## Pregnancy and CIH exposure

Although Chilean occupational health regulations explicitly prohibit pregnant women from working at altitudes exceeding 3,000 meters above sea level (1). There is no unified international legal framework governing pregnancy and occupational exposure to high altitudes. Nonetheless, international clinical and mountaineering guidelines generally advise against prolonged work or physical exertion at elevations exceeding 2,500–3,000 meters, particularly beyond 20 weeks of pregnancy and in the presence of risk factors such as anemia, hypertension, or a history of pregnancy complications (70, 71, 74). These recommendations are grounded in evidence indicating that hypobaric hypoxia has the potential to impair maternal–fetal oxygen exchange, elevate the risk of pre-eclampsia, and contribute to fetal growth restriction, especially in women who are not chronically acclimated to high altitude.

In the rare event that a pregnant worker is unintentionally exposed to CIH such as during unrecognized pregnancy or in countries without occupational restrictions, the combined hemodynamic load of pregnancy and the hypoxic stress of high-altitude work could exacerbate oxygen supply demand mismatches in the maternal circulation (44), a non-atherothrombotic ischemic event associated with increased morbidity and mortality. This pathophysiological mechanism is recognized in humans as a precipitant of Type 2 myocardial infarction. Clinical studies have shown that hypoxemia and anemia, both relevant to CIH, are independent predictors of worse outcomes in Type 2 MI. While no direct data exist for pregnant women under CIH, the overlap of these risk factors underscores the importance of early pregnancy detection, careful cardiovascular risk assessment, and adherence to preventive measures even in settings without legal prohibitions.

## Psychological dimension

Despite operating within a secure environment concerning gender equality and mutual sexual respect, women employed in the HA mining sector encounter a multifaceted set of psychological stressors that may augment cardiovascular risk. These encompass elevated cognitive demands, emotional stress associated with work–family conflicts, social isolation, and disturbances in sleep patterns. Such factors interact with neuroendocrine and autonomic systems, resulting in increased sympathetic activity and impaired BP regulation, which are indicative of cardiovascular dysregulation.

## Mental workload

A notable construct is mental workload, which pertains to the cognitive and emotional demands imposed by the work environment. Extended 12-h shifts, night work, and critical decision-making under pressure, particularly in hypoxic conditions, may lead to cognitive fatigue and impair executive functioning (45). According to the Demand-Control-Support model (46), Psychological strain intensifies when employees encounter high demands, limited autonomy, and minimal social support. These factors interact with neuroendocrine and autonomic systems, resulting in increased sympathetic activity and impaired BP regulation, which are characteristic of cardiovascular issues dysregulation.

## Emotional coping and perceived control

Women engaged in HA mining frequently encounter a conflict between their professional roles and caregiving responsibilities. Although this conflict is mediated by social factors, its psychological impacts are significant. Emotional tension, feelings of guilt, and diminished perceived control, especially among single mothers or individuals with limited familial support, can accumulate and manifest as chronic stress responses (47, 48). From a psychological standpoint, Bandura's theory of self-efficacy (49) highlights the protective role of perceived control in buffering these effects. Nevertheless, within male-dominated hierarchical settings, women frequently report diminished self-efficacy and limited decision-making autonomy (50).

These emotional stressors activate the hypothalamic-pituitary-adrenal (HPA) axis, thereby initiating the general stress response, or general adaptation syndrome (GAS). This leads to elevated cortisol levels, sympathetic overactivation, and vagal withdrawal. Such neuroendocrine imbalance disrupts cardiovascular homeostasis, fostering vascular inflammation, endothelial dysfunction, and heightened blood pressure variability, particularly during sleep and early morning hours.

## Circadian and neuroendocrine stress

Sleep disturbances represent among the most widespread psychological and physiological stressors encountered in high-altitude environments. High-altitude exposure adversely affects REM sleep and overall sleep efficiency, with a notably greater impact observed in women, who demonstrate increased susceptibility to these effects (51). Poor sleep quality is correlated with diminished parasympathetic activity and heightened cardiovascular stress (52). Recent evidence indicates that psychological stress at HA may also influence neuroendocrine pathways implicated in mood regulation and circadian physiology, notably the tryptophan–serotonin–melatonin axis, which is affected by hypoxic exposure. In women, sex-specific differences in serotonin synthesis and the modulatory role of estrogen amplify these modifications (51). Hypoxia-induced insomnia, coupled with diminished levels of serotonin and melatonin, may result in compromised thermoregulation and synaptic plasticity, thereby disrupting memory consolidation and emotional regulation. This sequence creates a neurobehavioral feedback loop in which stress, insomnia, and mood instability reinforce each other, exacerbating autonomic dysregulation and elevating cardiovascular workload over time.

Together, these psychological stressors facilitate cardiovascular dysregulation via persistent activation of the HPA axis, autonomic imbalance, and disruption of circadian rhythms. This neuroendocrine imbalance promotes vascular inflammation, endothelial dysfunction, and blood pressure dysregulation, which are fundamental mechanisms in the pathogenesis of cardiovascular disease (53). Within the GBM framework, these mechanisms emphasize the importance of assessing mental workload, coping capacity, and sleep quality as pivotal determinants of cardiovascular risk in women subjected to CIH.

## Social dimension

Psychosocial wellbeing, defined as the interaction among emotional, cognitive, and social functions, is integral to both mental and physical health. In high-altitude mining, social determinants of health include hierarchical workplace relationships, gendered role expectations, and the organization and social interpretation of work (54, 55). These environments are characterized by chronic intermittent hypoxia (CIH), extreme isolation, and rotational or fly-in, fly-out (FIFO) work schedules (4, 56). For women, these structural and cultural conditions may amplify the psychological stressors described above, particularly work–family conflict, limited decision-making autonomy, and reduced social support. While the physiological mechanisms, such as autonomic imbalance, vascular inflammation, and blood pressure dysregulation, are discussed in the psychological dimension, here we emphasize that the *origin* and *persistence* of these responses are strongly shaped by social context. Moreover, prolonged exposure to gendered workplace dynamics and inadequate health infrastructure may also impact reproductive health through hormonal disruptions and fertility-related issues (57, 58).

## Work-family balance

Women in mining work report higher levels of work-family conflict compared to men (59, 60). Extended work shifts, camp-based rotations, and FIFO arrangements may lead to extended periods of separation from children and family members. The logistical challenges of caregiving, whether involving bringing children into camp settings lacking childcare services or leaving them behind, can induce ongoing stress characterized by persistent feelings of guilt, anxiety, and emotional strain, particularly among single mothers who lack reliable caregiving support (47, 48, 61, 62). These stressors are not solely psychological; they trigger neuroendocrine responses derived from the general stress response, which elevate allostatic load and potentially increase cardiovascular strain over time.

## Gendered workplace dynamics

A secondary approach concerns the challenges related to workplace dynamics in predominantly male mining environments, which are generally characterized by isolated settings (63–66). Women frequently observe the necessity to exert exceptional effort to establish credibility, encounter limited access to decision-making roles, and endure scrutiny concerning their reproductive or maternal status (48, 50, 67). These conditions promote persistent hypervigilance, emotional suppression, and even burnout—factors directly correlated with elevated sympathetic tone, reduced HRV, and an increased risk of hypertension (55).

## Structural barriers to women's health

Certain infrastructural deficiencies and policy shortcomings may further exacerbate women's vulnerability to stress. Numerous mining sites are inadequately equipped with gender-specific protective equipment, menstrual hygiene amenities, and comprehensive healthcare support, particularly during pregnancy or menopause (54, 67). Even when women develop coping strategies, they often do so independently, in isolation, and without specialized institutional support. Such circumstances exert pressure to internalize and somatize systemic stressors as individual burdens, thereby increasing perceived helplessness and diminishing psychosocial resilience. Consequently, this may elevate cardiovascular risk (54, 67). Together, these social stressors do not operate independently; they interact dynamically with biological (e.g., hormonal) and psychological (e.g., sleep, mood) factors, collectively contributing to an elevated risk of persistent hypertension and cardiovascular dysregulation in women subjected to CIH.

## Prognostic implications of CIH in women

Prolonged exposure to chronic intermittent hypobaric hypoxia (CIH) may predispose women to cardiovascular changes that extend beyond immediate adaptation. Evidence from human studies involving rotating-shift workers subjected to high-altitude CIH indicates elevations in asymmetric dimethylarginine (ADMA), a biomarker of endothelial dysfunction and a predictor of hypoxia-induced pulmonary hypertension (72). These alterations, together with persistent hypoxaemia, surges in hypertension, and elevated sympathetic activity, facilitate a mismatch between oxygen supply and demand (68), a key pathophysiological mechanism in Type 2 myocardial infarction (44, 73).

Type 2 myocardial infarction, a non-atherothrombotic ischemic event, generally exhibits a poorer prognosis in women, particularly when hypoxaemia and anemia are present, conditions commonly observed in workers exposed to CIH. Furthermore, sex-specific factors such as iron deficiency, autoimmune disorders, hormonal fluctuations, and microvascular dysfunction may augment cardiovascular risk in this population women (69). Despite the absence of epidemiological data directly linking CIH to Type 2 myocardial infarction in women underscores the necessity for sex-specific preventive strategies, occupational monitoring, and longitudinal surveillance to facilitate early detection of cardiovascular deterioration among female workers at high altitudes.

## Integrative perspective

The interaction between hormonal, psychological, and social determinants under chronic intermittent hypoxia (CIH) reveals a complex, mutually reinforcing pathway to cardiovascular dysregulation in women. Hormonal status, whether marked by estrogen dominance, progesterone predominance, or androgenic profiles, modulates vascular tone, autonomic balance, and metabolic efficiency (27, 29). These biological responses are not isolated; they are amplified or attenuated by psychological stressors such as mental workload, circadian disruption, and emotional coping demands, which in turn activate neuroendocrine pathways (e.g., HPA axis) that further influence blood pressure regulation and vascular function (51, 53). Social stressors, including work–family conflict, gendered workplace dynamics, and structural barriers to health, provide the environmental context in which these physiological and psychological responses occur (61, 67). Within the GBM framework, these domains converge to shape cardiovascular adaptation or maladaptation, underscoring the necessity for integrated, context-specific preventive strategies in high-altitude occupational settings.

## Illustrative case scenario

Consider the case of a 38-year-old female mining operator working on a 7 × 7 shift rotation between sea level and 3,500 m.a.s.l., currently in the luteal phase of her menstrual cycle and using a combined oral contraceptive with moderate androgenic activity. During her high-altitude shift, elevated progesterone levels promote sympathetic activation, increasing heart rate and blood pressure, while hypobaric hypoxia imposes additional ventilatory and cardiovascular load (3, 27). Concurrently, she faces extended 12-h night shifts, high cognitive demands, and fragmented sleep due to circadian misalignment, compounding autonomic imbalance (51). Socially, she experiences persistent work–family conflict, being separated from her young children during rotation, and perceives subtle gender bias in task assignments (48, 67). In combination, these factors create a scenario of elevated allostatic load, reduced heart rate variability, and transient nocturnal hypertension, conditions that, if sustained over years, may increase her risk for endothelial dysfunction and Type 2 myocardial infarction (44, 69). This example illustrates how the GBM framework can be operationalized to identify risk profiles and guide targeted interventions in women exposed to CIH.

## Conclusion

Despite increasing interest in the health implications of CIH, most studies in the high-altitude mining populations have predominantly employed cross-sectional comparisons between individuals working at varying elevations. These research designs have seldom incorporated female participants and frequently neglect factors such as hormonal status, psychosocial burden, and occupational heterogeneity. Consequently, the fundamental mechanisms underlying cardiovascular risk in female workers subjected to real-world CIH exposure remain inadequately understood.

This mini-review presents the GBM as a conceptual framework for comprehending cardiovascular risk among women working at high altitudes. By integrating biological aspects (such as hormonal fluctuations and reproductive health), psychological factors (including emotional burden and sleep disturbances), and social dimensions (notably gendered workplace dynamics and caregiving roles), the GBM offers a contextually sensitive and equity-informed perspective for assessing cardiovascular adaptation under environmental stress.

Future research must progress beyond mere cross-sectional snapshots. There is an urgent need for longitudinal cohort studies to investigate how sex hormones, autonomic function, emotional stress, and structural inequities interact over time to influence the development of cardiovascular risks in women throughout their working lives. This is especially imperative given that female participation in high-altitude mining tasks is recent but rapidly increasing, while the long-term health implications remain largely unknown. The generation of sex- and gender-stratified physiological data, employing within-subject designs, and including targeted assessments of hormonal and psychosocial variables will be essential for advancing precision health methodologies and establishing equitable occupational health guidelines. Additionally, this model may inform occupational health policies and tailored surveillance strategies for women working in other high-altitude sectors such as astronomical observatories, military deployment, and border control.
